# On-chip graphene photodetectors with a nonvolatile *p*–*i*–*n* homojunction

**DOI:** 10.1038/s41377-025-01832-y

**Published:** 2025-07-07

**Authors:** Ruijuan Tian, Yong Zhang, Yingke Ji, Chen Li, Xianghu Wu, Jianguo Wang, Shuaiwei Jia, Liang Liu, Mingwen Zhang, Yu Zhang, Qiao Zhang, Zhuang Xie, Zhengdong Luo, Duorui Gao, Yan Liu, Jianlin Zhao, Zhipei Sun, Xuetao Gan

**Affiliations:** 1 Key Laboratory of Light Field Manipulation and Information Acquisition, and Shaanxi Key Laboratory of Optical Information Technology, School of Physical Science and Technology, Northwestern Polytechnical University, Xi’an, China; 2 Department of Electronics and Nanoengineering, Aalto University, Espoo, Finland; 3 State Key Discipline Laboratory of Wide Band Gap Semiconductor Technology, School of Microelectronics, Xi dian University, Xi’an, China; 4 State Key Laboratory of Transient Optics and Photonics, Xi’an Institute of Optics and Precision Mechanics, Chinese Academy of Sciences, Xi’an, China; 5 School of Microelectronics, Northwestern Polytechnical University, Xi’an, China

**Keywords:** Silicon photonics, Optical properties and devices

## Abstract

Graphene’s unique photothermoelectric (PTE) effect, combined with its compatibility for on-chip fabrication, promises its development in chip-integrated photodetectors with ultralow dark-current and ultrafast speed. Previous designs of on-chip graphene photodetectors required external electrical biases or gate voltages to separate photocarriers, leading to increased power consumption and complex circuitry. Here, we demonstrate a nonvolatile graphene *p–i–n* homojunction constructed on a silicon photonic crystal waveguide, which facilitates PTE-based photodetection without the need for electrical bias or gate voltages. By designing an air-slotted photonic crystal waveguide as two individual silicon back gates and employing ferroelectric dielectrics with remnant polarization fields, the nonvolatile *p*–*i*–*n* homojunction with a clear gradient of Seebeck coefficient is electrically configured. Hot carriers in the graphene channel generated from the absorption of waveguide evanescent field are separated by the nonvolatile *p–i–n* homojunction effectively to yield considerable photocurrents. With zero-bias and zero-gate voltage, the nonvolatile graphene *p*–*i*–*n* homojunction photodetector integrated on the optical waveguide exhibits high and flat responsivity of 193 mA W^−1^ over the broadband wavelength range of 1560–1630 nm and an ultrafast dynamics bandwidth of 17 GHz measured in the limits of our instruments. With the high-performance on-chip photodetection, the nonvolatile graphene homojunction directly constructed on silicon photonic circuits promises the extended on-chip functions of the optoelectronic synapse, in-memory sensing and computing, and neuromorphic computing.

## Abstract (web summary)

Graphene’s photothermoelectric effect and on-chip compatibility enable ultrafast, low-power chip-integrated photodetectors. We demonstrate a nonvolatile graphene *p*-*i*-*n* homojunction on a silicon photonic crystal waveguide, achieving efficient, bias- and gate-free photodetection with high responsivity, which is promising for advanced optoelectronics and neuromorphic computing.

## Graphical abstract

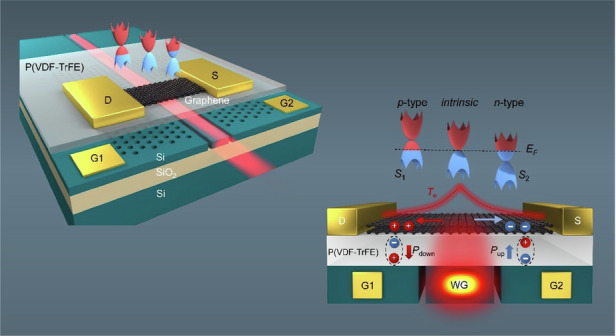

## Introduction

Photonic integrated circuits (PICs) are greatly expected to address the massive growth in data traffic of telecom, datacom, computing, sensing, etc^1–7^. To accomplish this, on-chip photodetectors, converting optical-to-electrical (O–E) signals, are desired to have attributes of ultrahigh bandwidth, high detectivity, low power consumption, low dark-current, and easy integration. Graphene has great advantages over other O–E converting materials for on-chip photodetectors with the above high merits^8^. As a semimetal hosting massless fermions, graphene represents one of the highest room-temperature carrier mobility of any material and extremely strong on-resonance light absorption from ultraviolet to terahertz range^9–11^. In the conversion of O–E signals, graphene has an intriguing photothermoelectric (PTE) effect beneficial from its unique hot carriers^12–15^. Because of the much weaker electron-phonon coupling than the electron-electron interaction, light-induced hot-carrier in graphene is so efficient that carrier multiplications are possible^13,14,16^. Combined with the record small heat capacity of graphene, the temperature of light-induced hot carriers could be increased substantially, driving the carrier diffusion to establish a large electrical potential difference via the thermal gradient. Hence, PTE-based graphene photodetectors could be operated without external bias to eliminate power consumption and dark current. Moreover, carrier cooling in graphene occurs on a picosecond timescale, enabling the PTE-based photodetection with ultrahigh bandwidth exceeding 200 GHz^9,17,18^. Finally, from the viewpoint of future large-scale production, graphene’s dangling-bond-free surface promises its wafer-scale integration with PICs without interface mismatch, for instance, in the back-end CMOS process.

Recently, on-chip graphene PTE photodetectors have been demonstrated by integrating them with photonic or plasmonic waveguides and resonators. To generate non-zero PTE current or voltage, there should be gradients of the Seebeck coefficient (Δ*S*) and hot-carrier temperature (Δ*T*_*e*_) across the graphene channel, which determine the PTE voltage in the form of *V*_PTE_ = Δ*S*Δ*T*_*e*_. Since the Seebeck coefficient *S* depends on the carrier doping level of graphene, junctions are expected to be involved over the graphene channel for realizing Δ*S*, which should be simultaneously designed to couple with the optical modes of the waveguides or resonators for obtaining Δ*T*. By integrating a metal-graphene Schottky junction with a silicon waveguide, we previously realized a PTE photodetection with a responsivity of 15 mA W^−1^ at zero bias and an ultrahigh bandwidth with only 1 dB degradation at 20 GHz^19^. However, the inevitable extra absorption loss by the metal electrode reduces the external quantum yield of the PTE photodetector. To eliminate this insertion loss, graphene *p*–*n* homojunctions were designed instead to couple with on-chip waveguides or resonators. For instance, graphene *p–n* homojunctions are integrated into silicon slot waveguides (or photonic crystal waveguides) to realize photodetection with an improved responsivity of 35 mA W^−1^ (48 mA W^−1^) at zero-bias and 3 dB bandwidth of 65 GHz (18 GHz)^20,21^. With the enhancement of a plasmonic mode, a micrometer-scale photodetector by coupling a graphene *p–n* homojunction with a metal-gap waveguide has been demonstrated with a responsivity of 12.2 V W^−1^ and a 3 dB bandwidth of 42 GHz without external bias^22^.

We note that, in these previous works, though the electrical bias across the graphene channel is not necessary, to ensure the considerable PTE voltage, two separated gate voltages have to be applied to the graphene layer over the selective areas to deterministically modify the doping levels and therefore yield Δ*S*. It complicates the operation and electrical circuits of the on-chip graphene photodetectors because two individual gate voltages have to be applied and maintained during the photodetection.

**Fig. 1:**
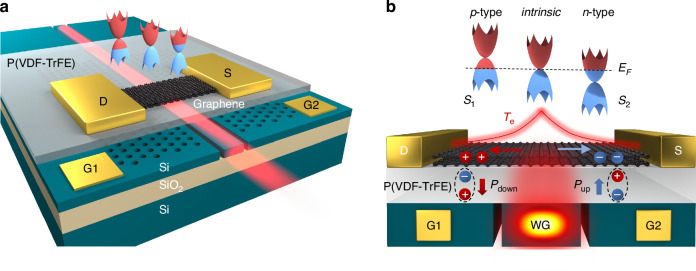
Schematic of the waveguide-integrated nonvolatile graphene *p*–*i*–*n* homojunction photodetector. **a** Schematic of the graphene *p*–*i–n* homojunction photodetector constructed on a PC waveguide with two air slots, showing optical mode propagating in the central waveguide and two separated back gates provided by the silicon PC slab with the P(VDF-TrFE) dielectric layer. **b** Gradients of Seebeck coefficient (Δ*S*) and hot-carrier temperature (*T*_e_) over the nonvolatile graphene *p–i–n* homojunction. A downward (upward) polarization field *P*_down_ (*P*_up_) of the P(VDF-TrFE) layer causes the graphene layer to be doped in *p*−type (*n*−type). Different doping levels of graphene gated by air-slotted PC waveguide result in the gradient of Seebeck coefficient Δ*S* = *S*_1_ *−* *S*_2_. The Fermi level of graphene is also provided for guiding the Seebeck coefficient variations related to the doping of graphene along the channel. Hot-carrier temperature *T*_e_ distributions of the graphene layer correspond to the guiding mode in the PC waveguide

Here, we introduce a strategy to realize chip-integrated graphene PTE photodetector without the requirement of maintained gate voltages by configuring nonvolatile *p*–*i–n* homojunction over the graphene channel. As schematically shown in Fig. 1a, a layer of poly(vinylidene fluoride-trifluoroethylene) (P(VDF-TrFE)) is spin-coated over a silicon photonic crystal (PC) waveguide, which is followed by the deposition of a graphene layer atop. The PC waveguide is split by two air slots into three electrically isolated regions. Because the employed silicon slab of the PC waveguide is slightly *p*-type doped, the two outside PC regions with arrays of air holes could function as two separated back-gate electrodes (G1, G2) of the top graphene layer. The top graphene layer is contacted with drain and source electrodes (D, S). By applying a gate voltage between the left (or right) silicon PC region and the top graphene layer through the P(VDF-TrFE) layer, the corresponding graphene section would be doped into *p*- or *n*-type depending on the direction of the gate voltage. Since there is no electrical signal applied on the central strip waveguide region, the corresponding top graphene section maintains intrinsic (*i*-type) without electrostatic doping. An electrostatically doped *p*–*i*–*n* homojunction is herein formed over the graphene channel, as shown in Fig. 1b. Note that, although there is slightly unintentional doping in the *i* region of graphene caused by the substrate doping and the transfer process, as demonstrated in many previous studies, it did not significantly impact the performance of *p*–*i*–*n* homojunction in our device (see the Supplementary Information for details). After that, the gate voltages are removed, while there are residual polarization fields (*P*_up_ or *P*_down_ state) at the top surface of P(VDF-TrFE) thanks to its ferroelectric property, which could hold the doping states of the interfacial graphene layer. Therefore, the graphene *p*–*i–n* homojunction is nonvolatile, i.e., its formation only requires one actuation of the pulsed gate voltages. This nonvolatile doping mechanism in graphene has also been demonstrated in the graphene-ferroelectric platform, such as LiNbO_3_ and PMN-PT crystal^23–26^. Consequently, varied specific distributions of Δ*S* over the graphene channel are held after their doping configurations^27–29^, which gets rid of the awkward operation of the maintained gate voltages.

In the designed PC waveguide split by two 100 nm wide air slots, the optical mode is well confined in the central strip waveguide due to the photonic bandgap of the outside PC structures^30^. The top graphene layer couples with and absorbs the evanescent field of the waveguide mode, which leaks out of the silicon slab and penetrates across the thin P(VDF-TrFE) layer. The absorbed light generates hot carriers over the graphene channel and subsequently induces a distribution of *T*_*e*_ governed by the waveguide mode profile^20,21^, as indicated in Fig. 1b. Combining with Δ*S* determined by the nonvolatile *p*–*i–n* homojunction, considerable PTE current could be achieved over the graphene channel without the applications of bias or gate voltage.

## Results

### Nonvolatile graphene *p–i–n* homojunction constructed on PC waveguide

**Fig. 2:**
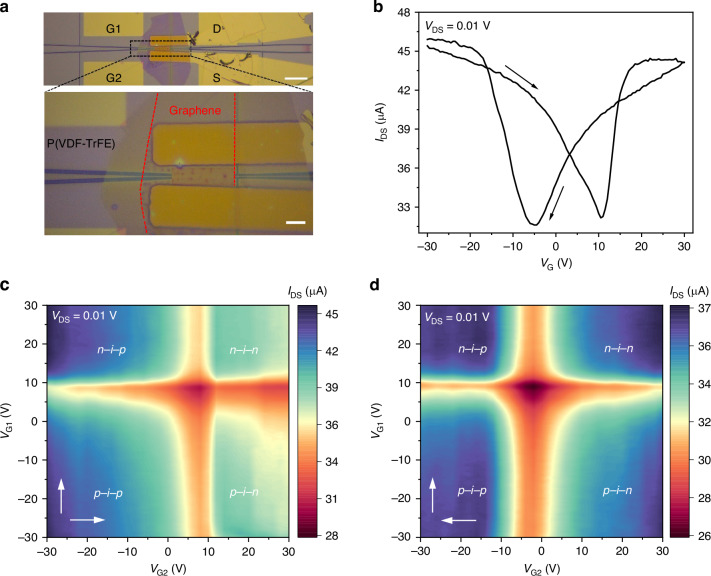
Electrical characteristics of nonvolatile graphene *p*–*i*–*n* homojunction constructed on the air-slotted silicon PC waveguide with the dielectric layer of P(VDF-TrFE). **a** Top panel: optical microscope image of a fabricated device. Scale bar, 50 μm. Bottom panel: the zoomed image of the active section of the device, consisting of graphene flake and P(VDF-TrFE) coated on the PC waveguide substrate. Scale bar, 10 μm. **b** Transfer curve of the graphene channel with the global gate voltages *V*_G_ = *V*_G1_ = *V*_G2_, and *V*_DS_ = 0.01 V. **c** Source-drain current *I*_DS_ map at *V*_DS_ = 0.01 V with varying *V*_G1_ from *−*30 V to 30 V, *V*_G2_ from *−*30 V to 30 V. **d** Source-drain current *I*_DS_ map at *V*_DS_ = 0.01 V with varying *V*_G1_ from 30 V to *−*30 V, *V*_G2_ from *−*30 V to 30 V, as indicated by the white arrows

Figure 2a shows the optical microscope image of the fabricated device. The fabrication flow and structure parameters are illustrated in Fig. S1 of Supplementary Information. The length of the waveguide-integrated graphene layer is ∼48.5 μm, ensuring a long light-graphene interaction. The output curve of the graphene channel between the source-drain current (*I*_DS_) and bias voltage (*V*_DS_) (see Fig. S2 of the Supplementary Information) is linear and symmetric, indicating good electrical contact.

Figure 2b shows the transfer curve (*I*_DS_ *−* *V*_G_) of the device when the two bottom silicon gates are connected together to act as a global back gate, i.e., *V*_G1_ = *V*_G2_ = *V*_G_. Due to the nonvolatile polarization field of the P(VDF-TrFE) film, an obvious hysteresis window appears by sweeping the gate voltages from *V*_G1_ = *V*_G2_ = −30 V to 30 V, then to −30 V. Specifically, when the gate voltage of −30 V is applied, the ferroelectric P(VDF-TrFE) layer is in the condition of its full downward polarization (*P*_down_) state. Thus, the accumulation of holes occurs over the graphene channel. The downward polarization state remains unchanged until an opposite voltage larger than the coercive condition is applied^29^. Upon sweeping the gate voltage toward zero and then to a positive direction, the hole current in the graphene channel persists. Until the gate voltage reaches positive coercive voltage, the direction of the ferroelectric polarization field of the P(VDF-TrFE) film starts to be reversed. The upward polarization (*P*_up_) state is fully compensated by electron accumulation over the graphene channel, and the electron current prevails as the gate voltage increases further toward +30 V. Furthermore, the electron current persists during the process of sweeping the gate voltage back to zero and downwards negative gate voltage. Until the gate voltage is close to negative coercive voltage, the polarization direction in the P(VDF-TrFE) layer switches back to the downward state, which re-establishes the hole current in the graphene channel. The observed transfer characteristics are attributed to the bipolar doping of the graphene layer and the nonvolatile polarization field of the P(VDF-TrFE) layer, as demonstrated by the hysteresis loop of the P(VDF-TrFE) layer (see the details in the Supplementary Information). This promises the construction of nonvolatile homojunction by setting the two silicon gates *V*_G1_ and *V*_G2_ individually.

Figure 2c, d presents the maps of |*I*_DS_| as a function of separated gates *V*_G1_ and *V*_G2_ at a fixed bias *V*_DS_ of 0.01 V with contrary *V*_G2_ sweeping directions. The four different doping configurations of *p*–*i–n*, *n–i–p*, *p–i–p*, *n–i–n* are clearly shown, as schematically illustrated in Fig. 1. The narrow mid-gaps between the *p–i–n*, *n–i–p*, *p–i–p*, and *n–i–n* regions located in the gate voltage levels of tuning graphene around the Dirac point, implying the rapid ferroelectric field switching process^29^. Due to the retention characteristics of the ferroelectric field, the scanning direction of the gate voltages marked by the white arrows affects the areas of different doping configurations and gate voltage levels of tuning graphene around the Dirac point. The memory window is also demonstrated in another device with similar current maps of |*I*_DS_| as a function of *V*_G1_ and *V*_G2_ (see Figs. S7 and S8 of the Supplementary Information). These current mapping figures contain at least 9 × 10^4^ voltage operations, demonstrating excellent endurance properties of the nonvolatile graphene *p–i–n* homojunction constructed on the silicon PC waveguide^31^.

**Fig. 3:**
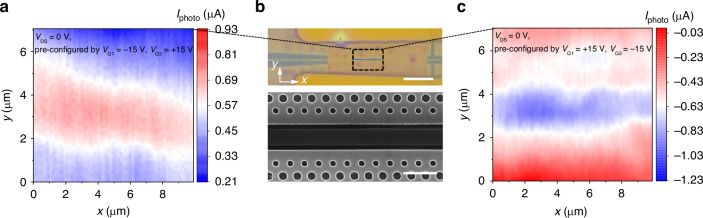
Spatially resolved photocurrent distribution of nonvolatile graphene *p*–*i*–*n* homojunction constructed on the air-slotted silicon PC waveguide with the dielectric layer of P(VDF-TrFE). **a** Scanning photocurrent map of the graphene *p*–*i*–*n* junction at *V*_DS_ = 0 V after a programming pulse gate voltage of *V*_G1_ = −15 V, *V*_G2_ = +15 V. **b** The corresponding scanning region is outlined by the black dashed line in the top panel. Scale bar, 10 μm. A zoomed view of the PC waveguide captured a part of the outlined region in the optical image of the top panel is shown in the bottom panel. Scale bar, 1 μm. **c** Scanning photocurrent map of the graphene *n*–*i*–*p* junction at *V*_DS_ = 0 V after a programming pulse gate voltage of *V*_G1_ = +15 V, *V*_G2_ = −15 V

To further demonstrate the nonvolatile and reconfigurable graphene *p–i–n* (*n–i–p*) homojunction and its capability of photodetection based on the PTE effect, we carry out the measurement of spatially resolved photocurrent by normally illuminating it with a focused laser beam (2 µm in diameter). There is no applied electrical bias *V*_DS_ = 0 V. The gate voltages are removed after the configuration of the homojunction. Figure 3a, c, respectively, display the scanning photocurrent maps at zero bias after the configuration pulses of (*V*_G1_ = −15 V, *V*_G2_ = +15 V) and (*V*_G1_ = +15 V, *V*_G2_ = −15 V). The corresponding scanning region of the device is marked by the black dashed line in Fig. 3b, which also shows the scanned electron microscope image of the slot PC waveguide. The inconsistencies of hole patterns in the SEM image are designed to enhance light-matter interaction by reducing the group velocity *v*_g_ of light in the waveguide (see the details in Fig. S9). As proposed in Fig. 1, after the programming pulses of *V*_G1_ = −15 (+15) V, *V*_G2_ = +15 (−15) V, the ferroelectric polarization field in the P(VDF-TrFE) layer gated by air-slotted PC waveguide respectively remains the upward and downward polarization state, which results in the *p* (*n*) doping for left side and *n* (*p*) doping for right side of the graphene channel. The middle region of the graphene channel is not gated by the gate voltage. As a result, in Fig. 3a, c, the photocurrent is generated by hot carriers diffusion with the gradients of Δ*T*_*e*_ and Δ*S*, following the relationship *V* = Δ*S*∙Δ*T*_*e*_, where *S* is the Seebeck coefficient of graphene in the corresponding dual-gated regions, and Δ*T*_*e*_ is the light-induced hot carrier temperature difference in the graphene channel. The direction of the photocurrent depends on the sign of the Seebeck coefficient gradient Δ*S*. Under a negative (positive) gate voltage, positive (negative) Seebeck coefficients are induced in the *p* (*n*) regions. In other words, for the *p*–*i*–*n* and *n*–*i*–*p* configurations across the graphene channel, the Seebeck coefficient gradients are Δ*S* and −Δ*S*, respectively. Consequently, the photocurrents exhibit opposite directions in Fig. 3a, c for the *p*–*i*–*n* and *n–i*–*p* homojunction configurations. This demonstrates that the nonvolatile graphene *p*–*i*–*n* (*n*–*i*–*p*) homojunction is successfully constructed. Benefiting from the nonvolatile and erasable properties of ferroelectrics, this waveguide-integrated graphene *p*–*i*–*n* homojunction could be arbitrarily written, reversed, and erased in a simple and low-cost way, which exhibits spectacular potential for applications in on-chip functional optoelectronic devices with low power consumption.

### Steady-state photoresponses of waveguide-integrated nonvolatile graphene *p*–*i–n* homojunction

**Fig. 4:**
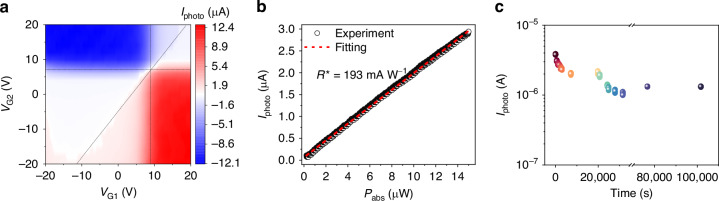
Steady-state photoresponses of the nonvolatile graphene *p*–*i*–*n* homojunction integrated on silicon PC waveguide with a laser at 1620 nm guided in the waveguide. **a** Measured photocurrent map at zero bias after the configurations with different gate voltages on G1 and G2. **b** Optical power dependence of the photocurrents at *V*_DS_ = 0 V after pulsed gate voltages of *V*_G1_ = −15 V and *V*_G2_ = +15 V to configure the graphene *p*–*i–n* homojunction. **c** Monitored photocurrents for 28 h after the removal of the gate voltages, displaying excellent endurance and retention performance

The achieved nonvolatile graphene *p–i–n* homojunction integrated on the PC waveguide is expected to support high-performance on-chip photodetections. To examine that, we couple light into the PC waveguide via the grating coupler at the end of the waveguide, as shown in Fig. S10a. A telecommunication band laser with a tunable wavelength range from 1500 nm to 1630 nm is used as the light source. The transmission spectrum of the device is shown in Fig. S10b of the Supplementary Information. With the applications of pulsed gate voltages (*V*_G1_, *V*_G2_) to configure the nonvolatile graphene *p–i–n* homojunction, the generated photocarriers by the absorption of waveguide evanescent field on the graphene channel are separated and collected by the D and S electrodes to yield a photocurrent, which is monitored with a high-precision electrical source-meter. With zero bias (*V*_DS_ = 0 V), the photocurrents measured at different configurations of gate voltages are shown in Fig. 4a. The clearly visible 6-fold pattern indicates that the PTE effect is the dominant conversion process^20–22,27^ in this waveguide-integrated graphene *p–i–n* homojunction photodetector. The PTE currents, due to the gradients of hot-carriers temperature and type-dependent Seebeck coefficient, cause excited carriers in the graphene to be separated and accelerated into opposite directions, without the need for an applied bias voltage.

To characterize the photoresponsivity of the waveguide-integrated graphene photodetector, Fig. 4b plots the measured photocurrents with varied absorbed optical powers of the incident laser at the wavelength of 1620 nm at *V*_DS_ = 0 V after the application of programming pulsed gate voltage of *V*_G1_ = −15 V and *V*_G2_ = +15 V. Benefiting from the remnant polarization field of the ferroelectric P(VDF-TrFE) layer, which maintains the doping type of the graphene channel, the light-induced carrier diffusion generates the photocurrent in the *p*–*i*–*n* configuration across the graphene channel. It shows a linear dependence, which is determined by the linear photocarrier separation and collection mechanism in the *p–i–n* junction configuration. The guided mode in the PC waveguide overlaps with the central intrinsic region of the top graphene *p–i–n* homojunction, as shown in the cross-sectional view of the electric field distribution in the PC waveguide (see Fig. S10c of the Supplementary Information). The absorption coefficient of the graphene layer on the guided mode is estimated as ∼0.17 dB μm^−1^ at the wavelength of 1620 nm as obtained from the mode simulations. It is basically consistent with the experimental measurement of ∼0.23 dB μm^−1^. Hence, the responsivity is estimated as *R* = *I*_photo_/*P*_abs_ = 193 mA W^−1^ at zero bias and zero-gate voltage. The corresponding internal quantum efficiency (IQE) is calculated as IQE = *R* × (*hc*/*qλ*) = 14.8%, where *R*, *h*, *c*, *q*, and *λ* are the responsivity, Planck’s constant, speed of light, electron charge, and wavelength, respectively. The high responsivity and IQE with zero bias are ascribed to the effective PTE effect endowed by the high-quality *p–i–n* homojunction^31^. The retention behavior of the photodetection performance is also evaluated. As shown in Fig. 4c, after the programming pulses of *V*_G1_ = −15 V and *V*_G2_ = +15 V, the photocurrent of the device at *V*_DS_ = 0 V remains nearly unchanged for more than 28 h, indicating the expected longer retention time. This retention property is attributed to the high crystallinity of the P(VDF-TrFE) layer, the high quality of graphene, and the clean interface between graphene and the ferroelectric layer, as demonstrated by our previous work^31^. This illustrates the good retention property of the waveguide-integrated nonvolatile graphene *p–i–n* homojunction, promising its easy implementation in future PIC applications without complex electrical circuits.

### Wavelength-dependent and high-speed photoresponses of the device

**Fig. 5:**
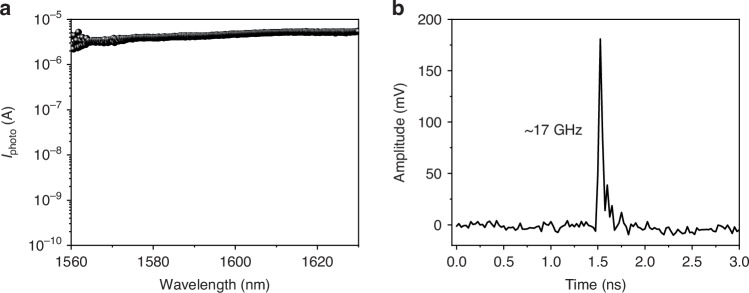
Wavelength-dependent and high-speed photoresponse of the waveguide-integrated nonvolatile graphene *p*–*i*–*n* homojunction photodetector at zero-bias after the configuration of *V*_G1_ = −15 V and *V*_G2_ = +15 V. **a** Wavelength-dependent photocurrent ranging from 1560 to 1630 nm. **b** Measured impulse response, showing a 3 dB bandwidth of 17 GHz limited by the employed oscilloscope

Leveraging the zero bandgap of graphene, it can absorb light over a broad spectral range. We evaluate the wavelength-dependent photoresponses of the waveguide-integrated graphene *p*–*i*–*n* homojunction photodetector, as shown in Fig. 5a. By tuning the wavelength of the laser coupled in the PC waveguide from 1560 nm to 1630 nm, the photocurrents of the configured nonvolatile graphene *p*–*i*–*n* homojunction are measured with the zero-bias and zero-gate voltages, showing an almost flat response. It promises a broadband operation in the telecommunication band. In future work, an edge coupler is preferred instead of a grating coupler to broaden the operation bandwidth of the proposed waveguide-integrated graphene photodetector. Benefiting from the ultrafast dynamics of the photogenerated hot carriers in graphene, the waveguide-integrated nonvolatile graphene *p*–*i*–*n* homojunction photodetector is expected to operate with high speed. We examine it with the measurements of impulse response. The impulse response of a photodetector reflects its ability to rapidly respond to a sudden change in light intensity, such as an optical pulse with a width of ~ps, supporting the high-speed demonstration of the photodetector. This method has been confirmed by other reported works^20,32^. A picosecond pulsed laser at the wavelength of 1550 nm is employed as the incident light source, which provides a train of 5 ps wide optical pulses. By sending the picosecond pulsed light into the device, the generated photocurrent pulses are monitored using a real-time oscilloscope. Figure 5b shows the measured impulse responses of the device at zero bias, which is configured by the pulsed voltages of *V*_G1_ = −15 V and *V*_G2_ = +15 V. The pulse relaxation time Δ*t* is extracted by counting the full-time width at half-maximum of the pulse peak. The result is Δ*t* ≈ 25 ps. The dynamic response bandwidth can be derived by the time-bandwidth product, giving *f*_3dB_ = 0.44/Δ*t* = 17 GHz. Even higher bandwidth is expected considering the mechanism of the ultrafast PTE effect in graphene, though our result is limited by the bandwidth of the employed oscilloscope. The bandwidth limitation of the employed oscilloscope is also confirmed by a commercial photodetector (KY-PRM-40G-I-SM). The device performance shows good consistency across multiple fabrications (see details in Figs. S11 and S12 of the Supplementary Information). The figures of merit of our photodetector, such as response speed and energy consumption, are superior to those of other state-of-the-art graphene photodetectors, as compared in Supplementary Table 1. A comprehensive comparison with waveguide-integrated photodetectors is also provided in Supplementary Table 2.

In the future, for scenarios involving motion detection, the on-chip nonvolatile graphene *p*–*i*–*n* photodetector can efficiently process in-memory sensing and computing by integrating high-performance image sensing, memory and computing capabilities^33^. This integration is facilitated by its ferroelectric nonvolatile characteristics, positive and negative photoresponses, and high-speed photoresponses. Furthermore, this system will consume zero electrical energy and reduce hardware overhead.

## Discussion

We have demonstrated nonvolatile graphene *p*–*i*–*n* homojunction directly constructed on silicon PC waveguide for high-performance photodetection. The doping type of the graphene channel is maintained by the residual polarization fields of the ferroelectric P(VDF-TrFE) dielectric layer between the top graphene and the bottom silicon gate. It makes the requirement for the external gate voltages obsolete, with a simplification of operation system circuits for the PICs. Relying on the conversion of incident light into an electrical signal via the PTE effect in graphene, considerable photocurrents could be obtained based on the Δ*T* governed by the mode profile of the waveguide and Δ*S* determined by the nonvolatile *p*–*i*–*n* homojunction. As a result, a high photoresponsivity of about 193 mA W^−1^ is obtained with the configuration of nonvolatile *p*–*i–n* graphene homojunction without bias and gate voltages. Due to the zero bandgap of graphene, the broadband photoresponse is illustrated by the flat responsivity in the range of 1560–1630 nm, which is limited by the cutoff wavelength range of the employed grating coupler. The ultrafast PTE effect in the graphene *p*–*i–n* homojunction also supports a high dynamic response bandwidth, which is illustrated by a value larger than 17 GHz as the highest bandwidth of our measurement instrument. The demonstrated device presents several competitive advantages over other waveguide-integrated photodetectors, particularly due to its bias-free and gate-free operation, resulting in low power consumption and negligible dark current, reducing the hardware overhead. Furthermore, the optoelectronic response of the nonvolatile graphene *p–i–n* homojunction integrated into silicon waveguide could also be utilized in various on-chip optoelectronic devices, such as optoelectronic synapse, in-memory sensing and computing, and neuromorphic computing, etc. In the future, its potential for large-scale integration offers significant promise for advancing artificial neural network-based intelligent sensory systems.

## Materials and methods

### Device fabrication

The PC waveguides, as well as the electrical isolation air-slots, were fabricated on a 220 nm thick silicon slab of a silicon-on-insulator substrate. The patterns were designed together in the same layout, which was defined in a 400 nm thick AR-P 6200.13 resist layer using the electron beam lithography. The patterns were next etched by inductively coupled plasma etching. After the transfer of the patterns to the silicon slab, the resist was chemically removed using *N*-methyl-2-pyrrolidone and piranha solution. The SEM image and simulated electric field distribution of the fabricated PC waveguide are shown in Fig. S9. Four electrically independent parts divided by the electrical isolation air slots in the silicon slab were used to support the electrodes of D, S, G1, and G2. The P(VDF-TrFE) (70:30 in mol %) thin film with a thickness of ∼150 nm as the dielectric layer was spin-coated on silicon PC waveguides, which was annealed at 135 °C in vacuum for 4 h. The 70:30 ratio for VDF and TrFE with excellent crystallinity and a dielectric constant of ten achieves high remnant polarization, which has been demonstrated by previously reported researches^34,35^. It can work well in an environment below 135 °C. The measured cross-sectional HR-TEM image of the P (VDF-TrFE) layer indicates high crystallinity (see Supplementary Information). The graphene layer was mechanically exfoliated onto the polydimethylsiloxane (PDMS, Gel-Pak) stamp using the Scotch tape method, which was then dry transferred onto the P(VDF-TrFE) film with the assistance of a precise alignment system^36,37^. The thickness was roughly identified by optical contrast and then confirmed by Raman spectroscopy and atomic force microscopy (see Fig. S4 of the Supplementary Information). Next, photolithography-defined Au electrodes with a thickness of 50 nm were picked by PDMS and released accurately onto the graphene layer as the D and S electrodes using the transfer-printing technique^38–40^. A detailed fabrication flow of nonvolatile graphene *p–i*–*n* homojunction gated by air-slotted PC waveguide is also schematically shown in Fig. S1 of the Supplementary Information.

### Electrical and optoelectronic measurements

Electrical and optoelectronic measurements of the fabricated waveguide-integrated nonvolatile graphene *p*–*i*–*n* homojunction photodetector were carried out with a semiconductor parameter analyzer (PDA FSpro) at room temperature in ambient conditions. A tunable laser in the telecom band (TUNICS T100S-HP) was used as the light source, which was launched into an optical fiber and aligned to the input grating coupler of the PC waveguide with a six-axis micromanipulator. The transmission optical powers of the PC waveguide were monitored from the other grating coupler by an optical power meter. High-frequency photoresponses were performed by the impulse response measurements. A train of optical pulses (with a pulse width of 5 ps and a repetition rate of 20 MHz) at the wavelength of 1560 nm generated by an optical parametric oscillator was coupled into the device via the grating couplers. The impulse photocurrents generated from the fabricated photodetector were extracted by using an RF-GS probe (MPI T26A GS150). An electrical amplifier (Mini-circuits, ZKLNA-020GHz) was used to amplify the extracted impulse photocurrents. Finally, the amplified electrical signal was transmitted into the oscilloscope (Lecroy_SDA 825Zi-A), and the impulse response was measured.

## Supplementary information

On-chip graphene photodetectors with a nonvolatile p−i−n homojunction

## Data Availability

All data used in this study are available from the corresponding authors upon reasonable request.
