# Supplementary material for: On-chip graphene photodetectors with a nonvolatile p–i–n homojunction
Source: Light Sci Appl. 2025 Jul 7;14:238. doi: 10.1038/s41377-025-01832-y (PMC12234681; doi:10.1038/s41377-025-01832-y)
Supplement: Supplementary file 1 — On-chip graphene photodetectors with a nonvolatile p−i−n homojunction [file 41377_2025_1832_MOESM1_ESM.docx]

**Supplementary Information for**

**On-chip graphene photodetectors with a nonvolatile *p*−*i*−*n* homojunction**

Ruijuan Tian^1,2^, Yong Zhang^3^, Yingke Ji^1^, Chen Li^1^, Xianghu Wu^1^, Jianguo Wang^1^, Shuaiwei Jia^4^, Liang Liu^5^, Mingwen Zhang^1^, Yu Zhang^1^, Qiao Zhang^1^, Zhuang Xie^4^, Zhengdong Luo^3^, Duorui Gao^4^, Yan Liu^3^, Jianlin Zhao^1^, Zhipei Sun^2,^*, Xuetao Gan^1,5,^*

1. Key Laboratory of Light Field Manipulation and Information Acquisition, and Shaanxi Key Laboratory of Optical Information Technology, School of Physical Science and Technology, Northwestern Polytechnical University, Xi’an 710129, China
2. Department of Electronics and Nanoengineering, Aalto University, Espoo 02150, Finland
3. State Key Discipline Laboratory of Wide Band Gap Semiconductor Technology, School of Microelectronics, Xi dian University, Xi’an 710071, China
4. State Key Laboratory of Transient Optics and Photonics, Xi’an Institute of Optics and Precision Mechanics, Chinese Academy of Sciences, Xi’an 710119, China
5. School of Microelectronics, Northwestern Polytechnical University, Xi’an 710129, China

**Email:* [zhipei.sun@aalto.fi](mailto:zhipei.sun@aalto.fi); [xuetaogan@nwpu.edu.cn](mailto:xuetaogan@nwpu.edu.cn)

**
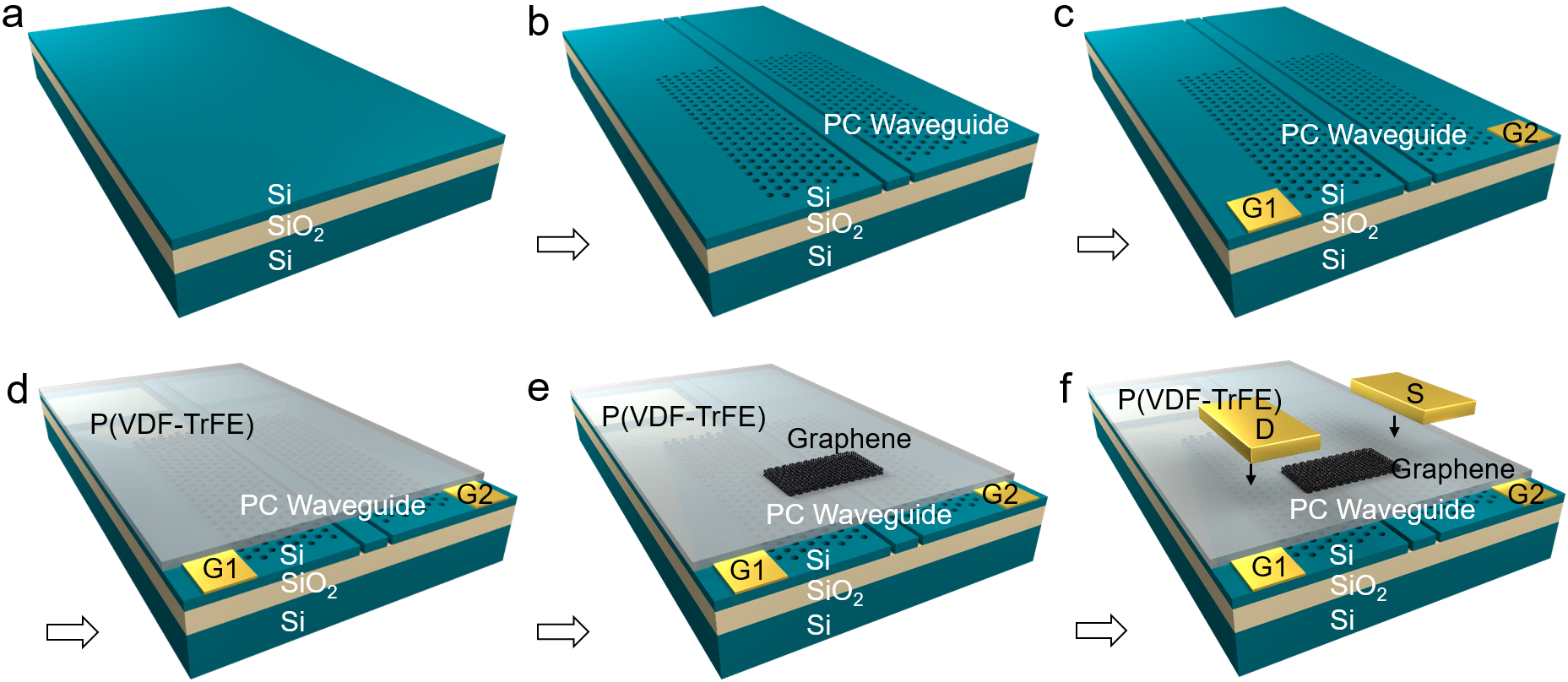
**

**Figure S1** Fabrication flow and structure parameters of the device. **a** A 220 nm thick silicon-on-insulator wafer was prepared for device fabrication. **b** PC waveguide is fabricated on the 220 nm thick silicon-on-insulator wafer using the techniques of electron beam lithography and inductively coupled plasma etching. The air-holes of the PC pattern have a lattice constant of 420 nm and a radius of 110 nm. The waveguide is formed by missing one line of air-holes. At the boundary between the air-hole region and the central line-defect, there are two 100 nm wide air-slots, which leave the middle waveguide with a width of 520 nm. **c** D, S, G1, and G2 electrodes are respectively defined in the four electrically independent parts of the silicon slab divided by the electrically isolated air-slots. **d** P(VDF-TrFE) (70:30 mol%) ferroelectric polymer was prepared by spin coating on the top of the silicon slab. After that, the P(VDF-TrFE) films were annealed at 135 °C for four hours to improve the crystallinity. **e** Few-layer graphene was exfoliated and then transferred on the P(VDF-TrFE) film coated on the electrically isolated air-slots PC waveguide. The thickness of the graphene layer was roughly identified by optical contrast, and later confirmed using atomic force microscopy and Raman spectrum. **f** Finally, Au electrodes with photolithography-defined shape were transferred on both sides of the graphene for drain and source contact.


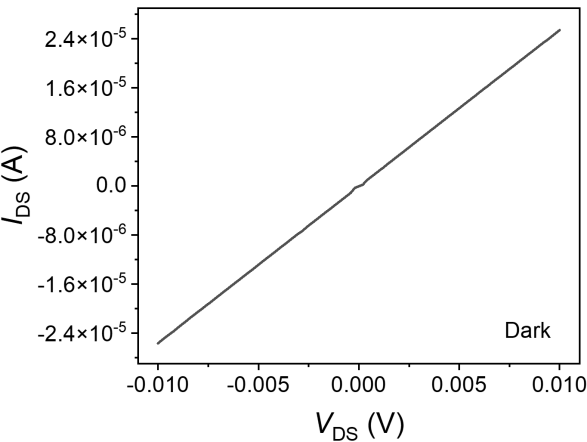


**Figure S2** Output characteristic (*I*_DS_–*V*_DS_) curve of waveguide-integrated nonvolatile graphene *p*–*i*–*n* junction at *V*_G1_=*V*_G2_=0 V.


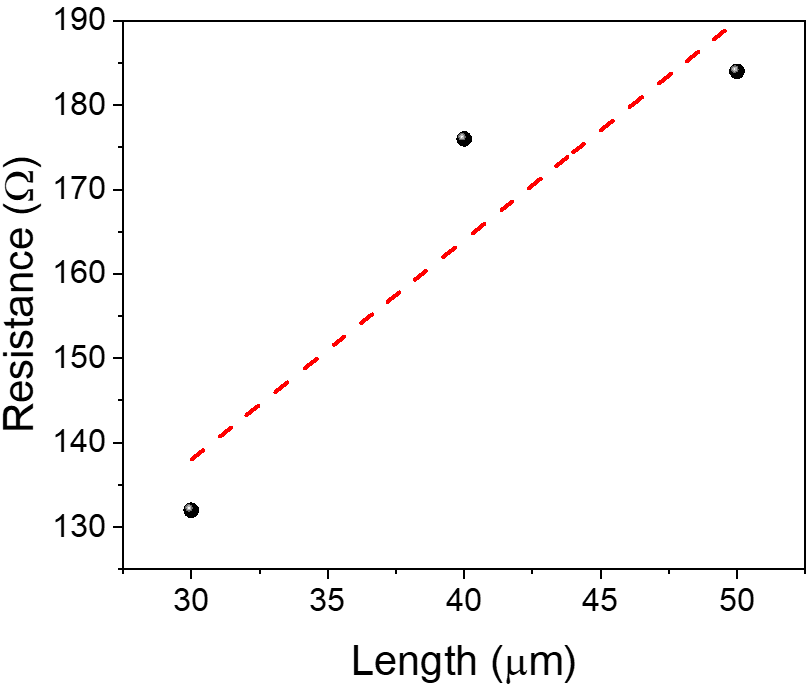


**Figure S3** Resistance of the graphene device, extracted from *I*_DS_-*V*_DS_ curves with different channel lengths at a width of 0.5 μm, plotted against the channel lengths. By fitting the equation *R*_total_= 2*R*_contact_+*R*_sheet_⋅*L*/*W*, we extract the contact resistance of *R*_contact_= 60 ± 42 Ω.

**
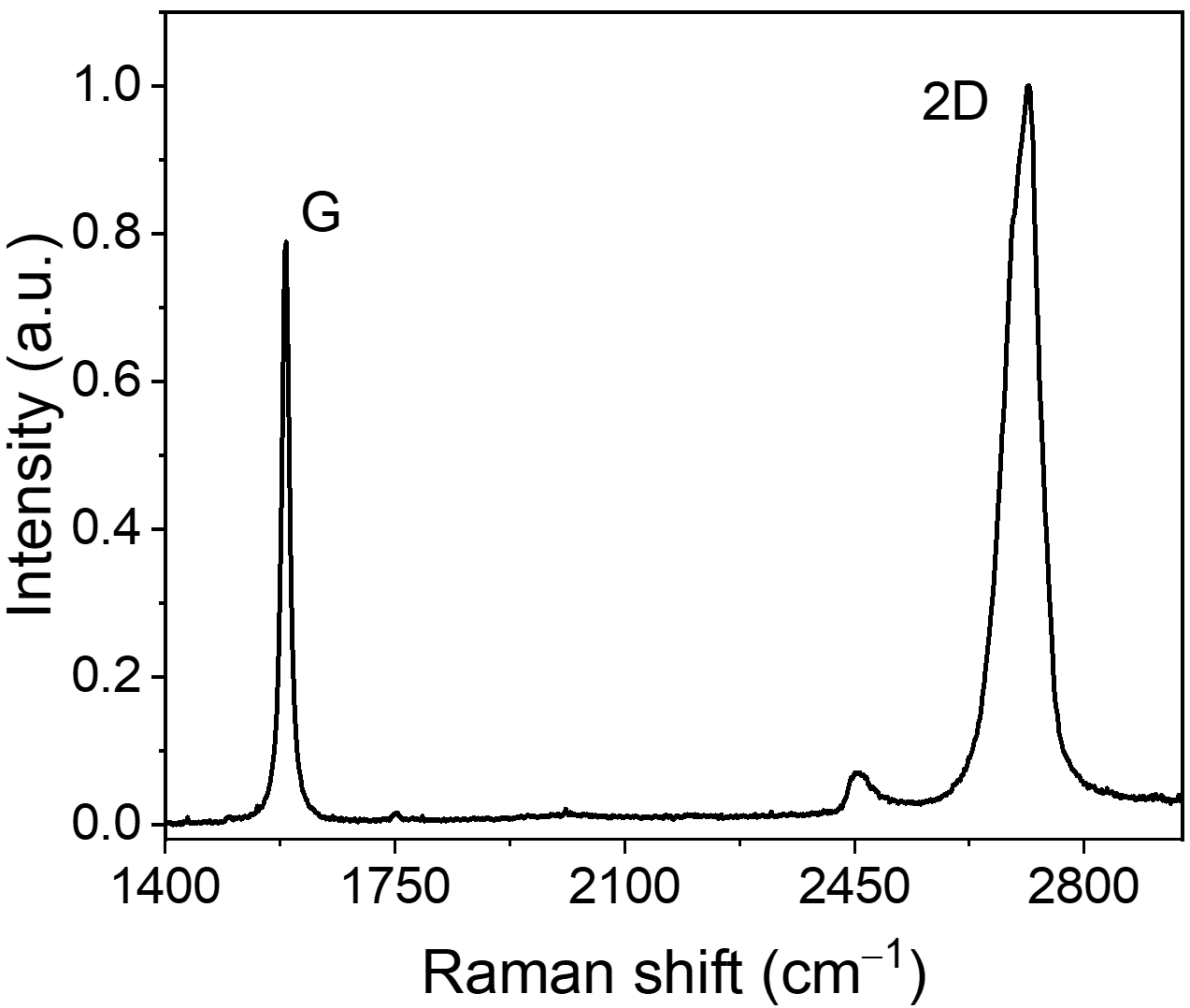
**

**Figure S4** Raman spectra of the fabricated device in the maintext. Raman spectrum of the graphene shows two characteristic peaks: the G peak at ~1582.7 cm^-1^, and the 2D peak at ~2703.8 cm^-1^. It shows the bilayer graphene thickness, which is comprehensively determined by the Raman characteristic of G peak intensity, G and 2D peak intensity ratios and 2D peak type^S1^.


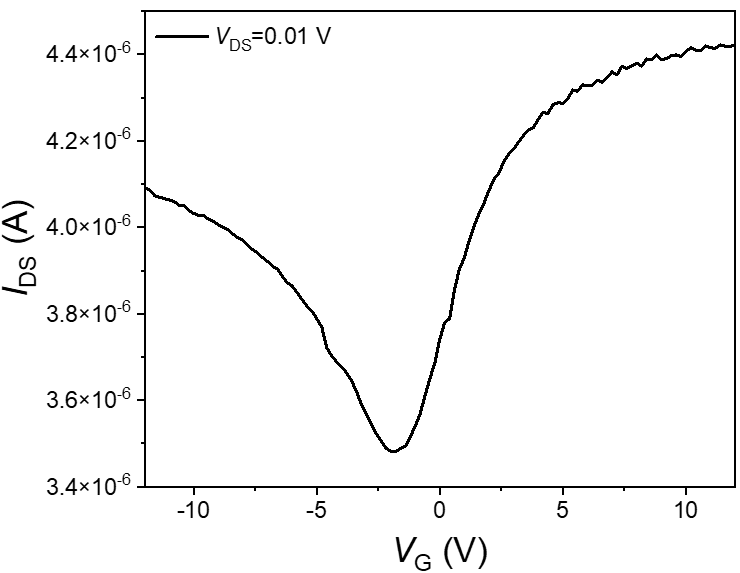


**Figure S5** Transfer curves for the graphene FET device fabricated using the mechanical exfoliation method and micro-transfer printing.

Raman spectroscopy and transfer curve (Fig. S4 and Fig. S5) demonstrate that the graphene in the middle region of the channel is nearly intrinsic, with minimal doping. This slightly unintentional doping does not significantly impact the performance of the *p*–*i*–*n* homojunction in our device. The photodetector’s overall performance, particularly its high responsivity and response speed, reflects strong optical absorption and highly efficient photoelectric conversion within the *p*–*i*–*n* graphene homojunction.


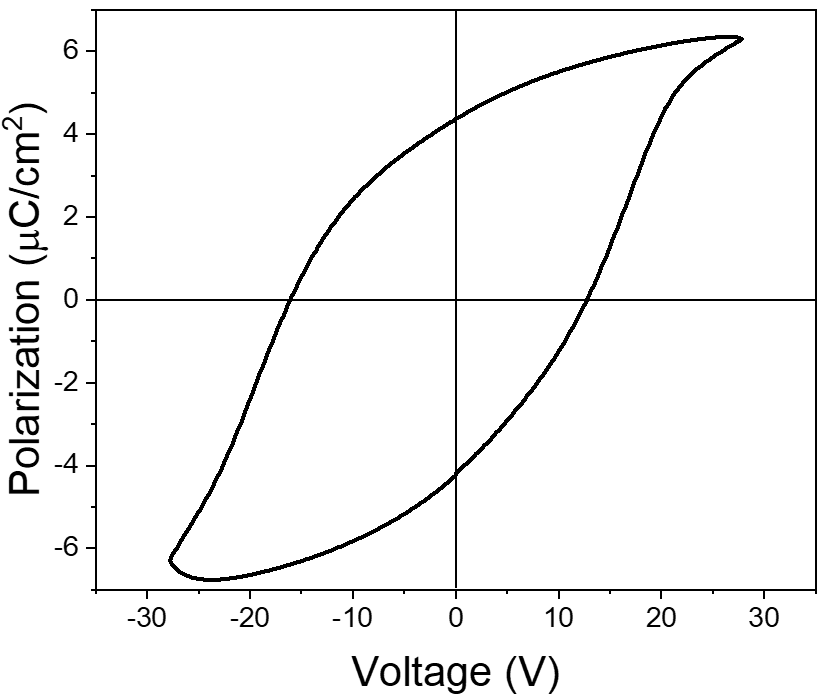


**Figure S6** Ferroelectric hysteresis loop of the Au/P(VDF-TrFE)/Au capacitor, measured at 500 Hz.

Figure S6 presents the ferroelectric hysteresis loop of an Au/P(VDF-TrFE)/Au capacitor, indicating that the polarization direction of the ferroelectric polymer can be defined and reversed using an applied electric field. The intersection of the spontaneous polarization loop at zero electric field corresponds to the remnant polarization (*P*_r_), that is, the electric polarization remains in the material after the removal of the external electric field. From this hysteresis loop, the value of *P*_r_ is determined to be 4.37 μC cm^-2^. For the coercive field (*E*_c_), which is the minimum electric field required to switch the full remnant polarization, ~12 V is observed in the hysteresis loop of the P (VDF-TrFE) layer.

According to the hysteresis loop of P(VDF-TrFE) layer (Fig. S6), we can explain the unique transfer curves in Fig. 2b more intuitively. The hysteresis observed in the transfer curve directly corresponds to the polarization switching behavior of the P(VDF-TrFE) layer. The "memory effect" in the ferroelectric material, as its ability to retain polarization even when the gate voltage is removed, creates the distinct forward and backward sweeps in the transfer curve. The ferroelectric polarization states (*P*_up_ and *P*_down_) control the accumulation of charge carriers (electrons or holes) in the graphene channel, leading to the observed hysteresis in the current as the gate voltage is swept. The coercive voltages of the P(VDF-TrFE) define the points where the polarization switches, switching from hole to electron in the graphene channel, which exhibits in the hysteresis loop and transfer curves.


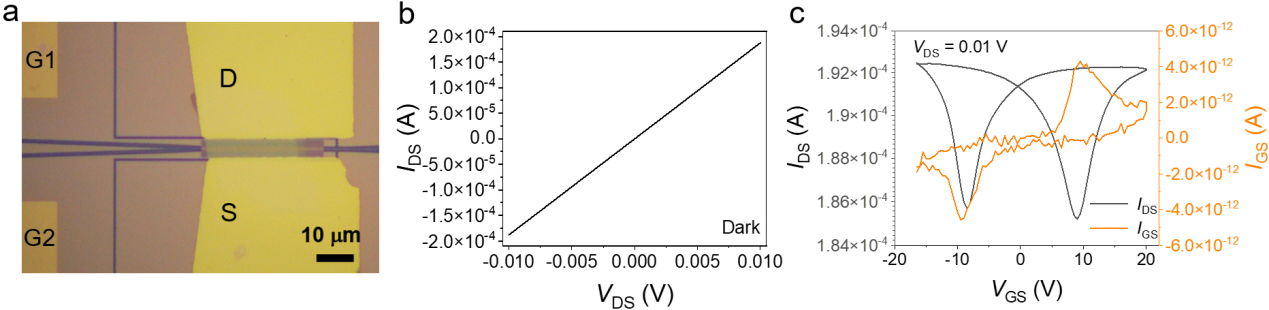


**Figure S7** Electrical characteristics of another waveguide-integrated graphene *p*–*i*–*n* homojunction photodetector (marked as Device B). **a** Optical micrograph of the device. **b** *I*_DS_-*V*_DS_ curve with *V*_G1_=*V*_G2_= 0 V. **c** *I*_DS_-*V*_GS_ and *I*_GS_-*V*_GS_ curves when the bottom gates are connected. Specifically, the two bottom silicon gates are connected together to act as a global back gate, i.e., *V*_G1_=*V*_G2_=*V*_G_, which don’t have other parallel path to contribute the gate leakage current. It has similar symmetrical memory windows with the device of the main text. The dark current level for device B is slightly different from that in the device discussed in the main text because of the different graphene thicknesses. However, the similar symmetrical memory windows and on- and off-current states demonstrate the robust ferroelectric polarization field and gate tunability, which are the key factors influencing the device's performance. This reinforces the confidence of device’s reproducibility.


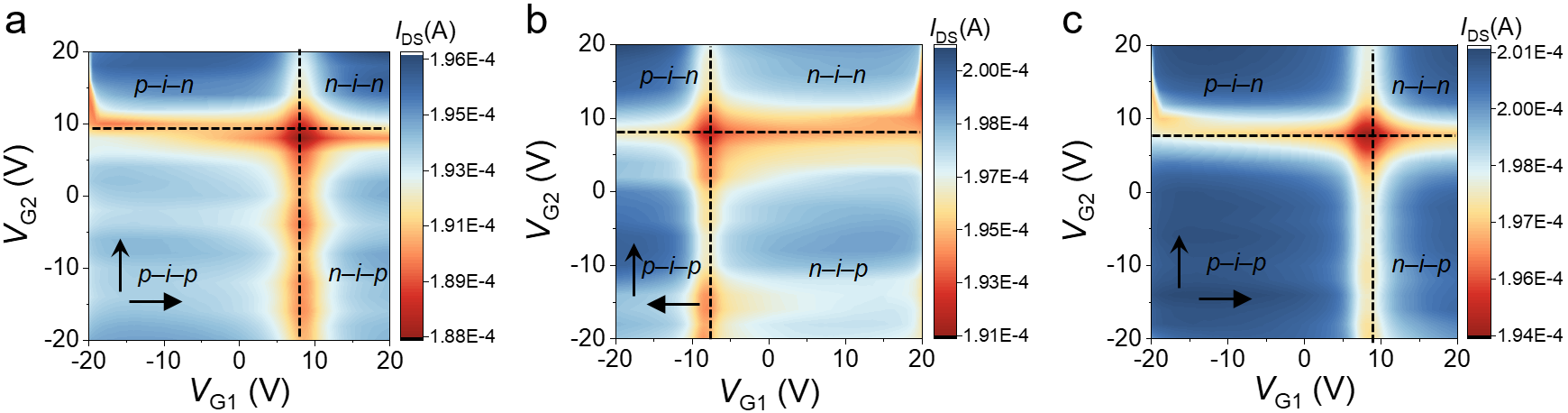


**Figure S8** Source-drain current *I*_DS_ as a function of two independent gate voltages *V*_G1_ and *V*_G2_ at a fixed source-drain bias *V*_DS_ of 0.01 V of Device B. The sweep direction of gate voltage is indicated by the arrows. **a** Sweep direction of *V*_G2_ is from –20 V to 20 V while *V*_G1_ sweeps from –20 V to 20 V. **b** Sweep direction of *V*_G2_ is from –20 V to 20 V while *V*_G1_ sweeps from 20 V to –20 V. **c** Sweep direction of *V*_G2_ is from –20 V to 20 V while *V*_G1_ sweeps from –20 V to 20 V again. Those three current mapping figures with similar on- and off-current states contain more than 9×10^4^ voltage operations, demonstrating excellent endurance property of the device.

**
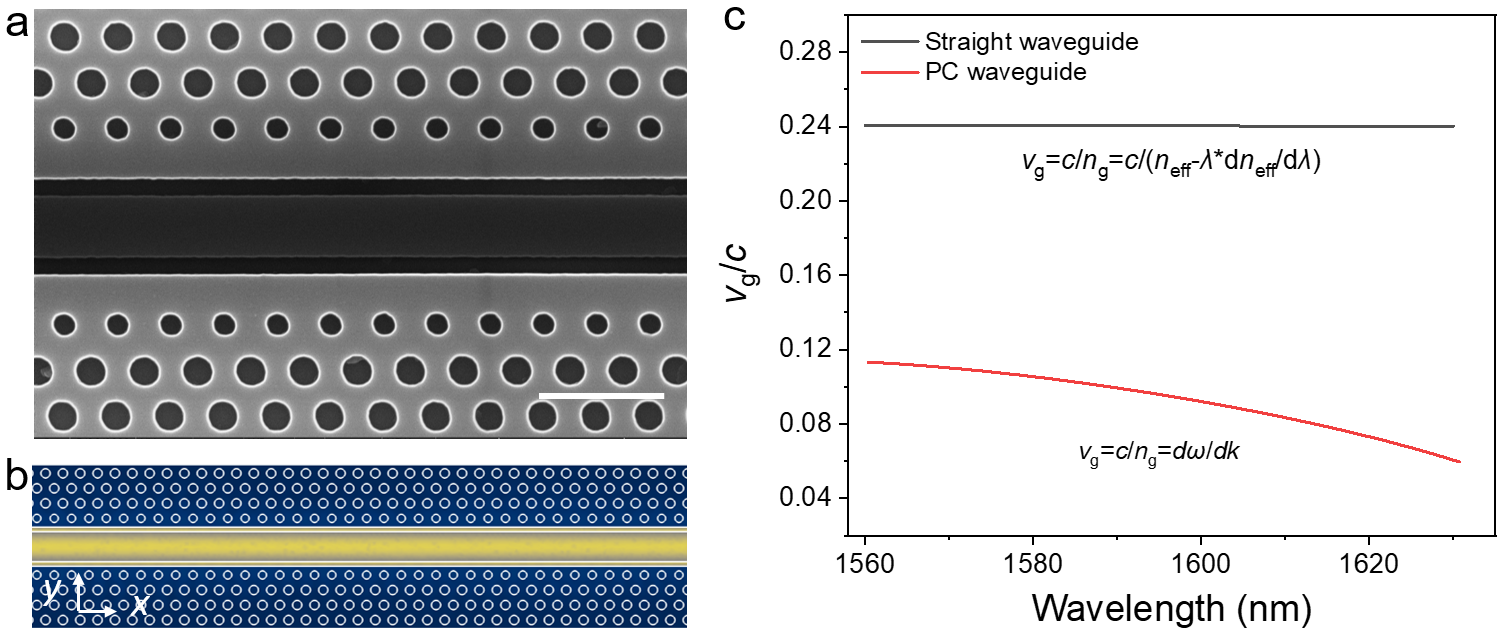
**

**Figure S9** **a** SEM image of the PC waveguide with two air-slots. Scale bar, 5 μm. **b** Simulated electric field distributions of the guided mode in the PC waveguide. **c** Group velocity of the optical field transmitted in the PC waveguide compared to that in the straight waveguide.

To analyze the optical behavior of the photonic crystal (PC) waveguide, we compare the group velocity (*v*_g_) of the optical field in both the PC waveguide and a straight waveguide. The group velocity (*v*_g_) of light in a waveguide is critical in determining light-matter interactions. A reduction in group velocity enhances light-matter interactions by extending the effective interaction time between the optical field and the material. Specifically, as the group velocity decreases, light propagates more slowly through the waveguide, thereby increasing the duration of light-matter interaction.

We next simulate the PC waveguide using the finite element method. Figure S9a shows the inconsistent hole patterns of the PC waveguide. The simulated electric field distributions of the guided mode in the PC waveguide are shown in Fig. S9b. From the simulation, we also obtain the group refractive index (*n*_g_) of light in the PC waveguide. In the PC waveguide, the group velocity (*v*_g_) can be expressed as *v*_g_ = *c*/*n*_g_ = d*ω*/d*k*, where *ω* is the angular frequency of the optical field, and *k* is the wave vector. In a conventional optical waveguide (such as a straight waveguide), the group velocity can be described using the effective refractive index approach: *v*_g_ = *c*/*n*_g_ = *c*/(*n*_eff_ - *λ*·d*n*_eff_/d*λ*), where *c* is the speed of light in a vacuum, *n*_eff_ is the effective refractive index of the waveguide mode, and *λ* is the light wavelength. Based on this, we plot the group velocity (*v*_g_) for both the PC waveguide (red line) and the straight waveguide (black line) across the operational wavelength range, as shown in Fig. S9c. The results demonstrate that the group velocity in the PC waveguide is significantly lower than that in the straight waveguide. This reduced group velocity in the PC waveguide increases the light-matter interaction time, thereby enhancing light-matter interaction.


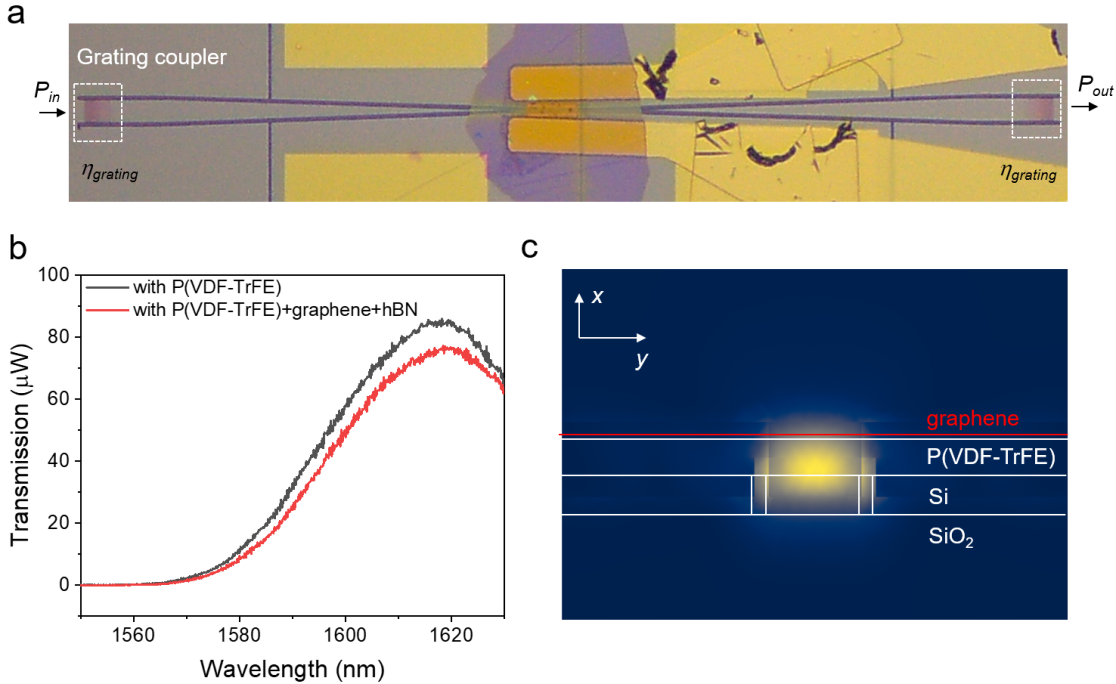


**Figure S10** Optical characterizations of the PC waveguide. **a** Schematic diagram of the proposed waveguide-integrated nonvolatile graphene *p−i−n* homojunction photodetector under the testing system. The grating couplers were employed for coupling light to one side of the PC waveguide and collecting light from the other side of the PC waveguide. **b** Transmission spectra of the PC waveguide covered by P(VDF-TrFE) layers before (black line) and after (red line) transferring graphene and h-BN layers. **c** Cross-sectional view of simulated electric field distributions of the guided mode in the PC waveguide integrated with the P(VDF-TrFE) and graphene layer.

To measure the optical absorption of the integrated graphene on the PC waveguide, the transmission spectra are measured before and after the graphene integration. The schematic diagram of the proposed device is shown in Fig. S10a. There are two grating couplers at the two ends of the PC waveguide. The transmission spectra are measured by sending the laser light from a tunable laser into one grating coupler and collecting the transmission power from the other grating coupler. Figure S10b shows the transmission spectra of the fabricated silicon PC waveguide covered by P(VDF-TrFE) layer before (black line) and after (red line) graphene device preparation. After the device fabrication is finished, the power of transmission spectrum decreases due to the absorption of the few-layer graphene. Here, we calculate the absorption power of graphene by the following method, which is confirmed by our previous work^S2^.

The output power of the waveguide coated with P(VDF-TrFE) film can be defined as

*P*_out1_= *P*_in_×*η*_g_×*η*_g_×(1-*α*_1_) (1)

where *P*_in_ is the power of incident laser light and *η*_g_ is the grating coupler efficiency.

The output power of the finished device can be defined as

*P*_out2_= *P*_in_×*η*_g_×*η*_g_×(1-*α*_3_) (2)

where *α*_1_ is the waveguide and P(VDF-TrFE) film loss efficiency, *α*_3_ is waveguide, P(VDF-TrFE) film and graphene absorption efficiency. Thus, the absorption power of graphene can be expressed as

*P*_abs_ = *P*_in_×*η*_g_×*α*_2_  (3)

where *α*_1_ is the graphene loss efficiency obtained by the difference of *α*_3_ and *α*_2_.

In order to obtain the coupling efficiency of the grating coupler, the transmission spectra before and after transferring graphene device are overlapped, and the coupling efficiency of the grating with spin-coated P(VDF-TrFE) is extracted as 22.4% at the wavelength of 1620 nm. The absorption coefficient of the graphene layer on the waveguide mode is obtained as ~0.23 dB μm^-1^ at the wavelength of 1620 nm.

The theoretically anticipated absorption coefficient of graphene on the waveguide is also calculated using FEM software COMSOL. The waveguide supports a single, quasi-TE mode as expected, with the maximum electric field in *x*-direction. The mode profile is shown in Fig. S10c and the theoretically anticipated absorption of 0.17 dB μm^–1^ is consistent with the experimentally extracted absorption of 0.23 dB μm^–1^ for the few-layer graphene.

**
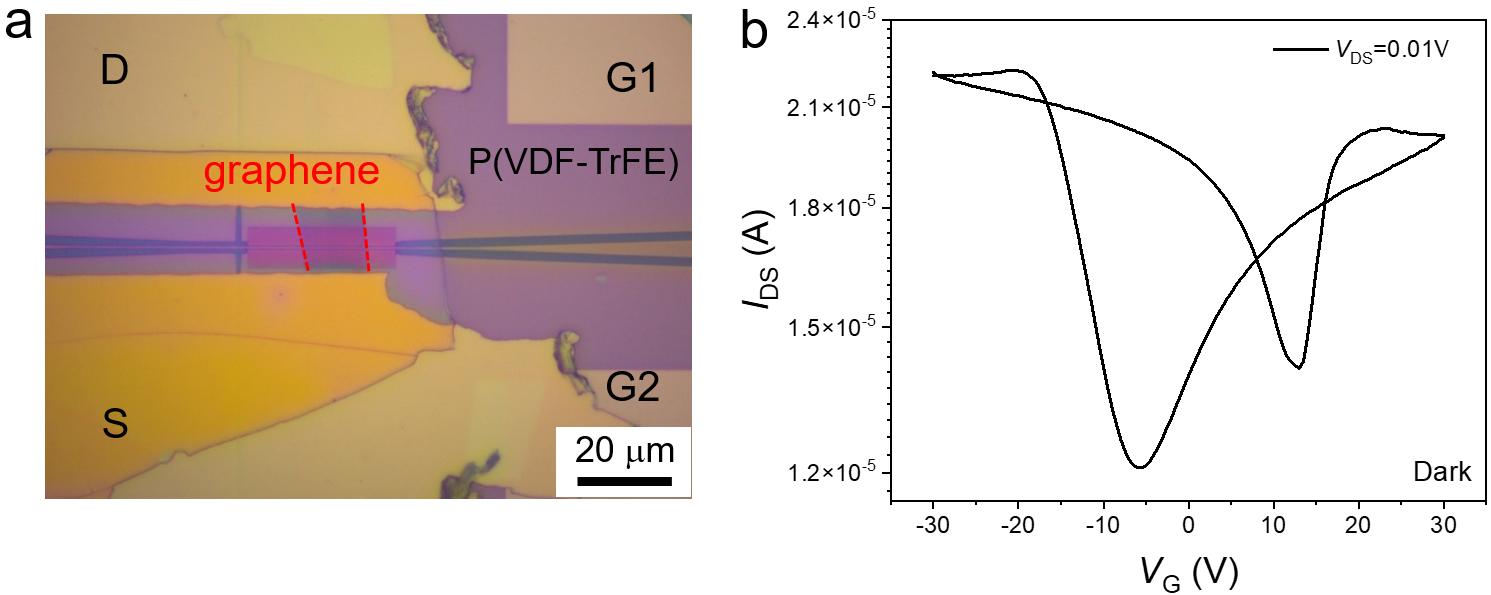
**

**Figure S11** Electrical characteristics of another waveguide-integrated graphene *p*–*i*–*n* homojunction photodetector (referred to as Device C). **a** Optical micrograph of the device. **b** *I*_DS_-*V*_GS_ curves when the bottom gates are connected, showing similar symmetrical memory windows to those of the device described in the main text.

**
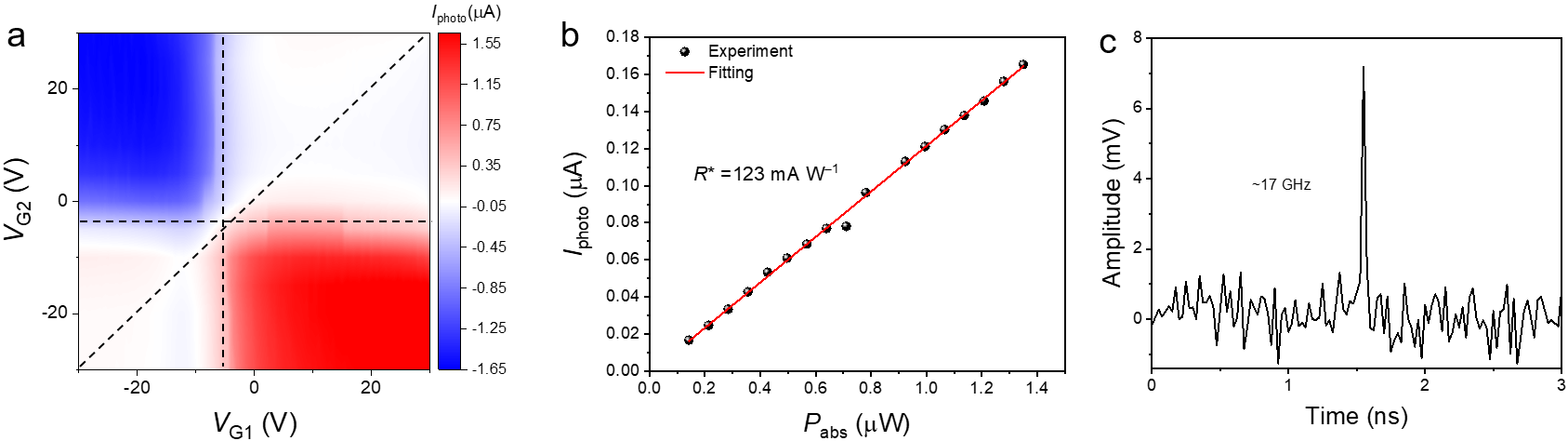
**

**Figure S12** Photoresponses of another waveguide-integrated graphene *p*–*i*–*n* homojunction photodetector (referred to as Device C) with a laser at 1620 nm guided in the waveguide. **a** Measured photocurrent map at zero-bias after the configurations with different gate voltages on G1 and G2. **b** Optical power dependence of the photocurrents at *V*_DS_ = 0 V after pulsed gate voltages of *V*_G1_= −15 V and *V*_G2_= +15 V to configure the graphene *p*−*i*−*n* homojunction. All of these devices shows good repeatability and reproducibility of the device. **c** Measured impulse response, showing a 3 dB bandwidth of 17 GHz limited by the employed oscilloscope.


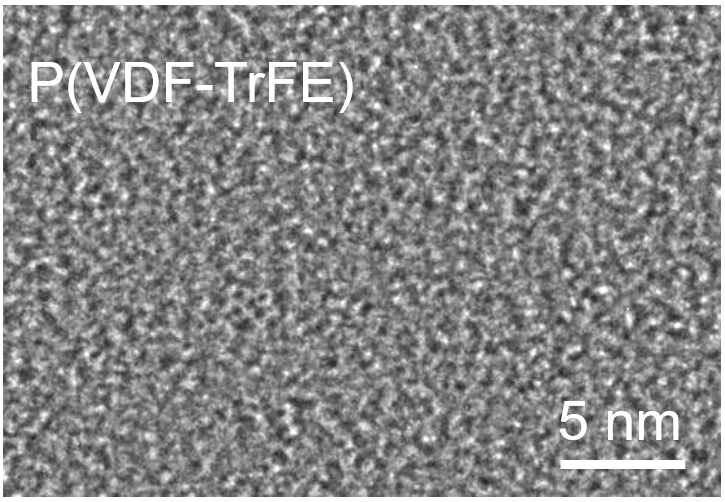


**Figure S13** Cross-sectional HR-TEM images of the P (VDF-TrFE) layer. This indicates high crystallinity P(VDF-TrFE) ferroelectric layer.

The following table serves as a comparison of the waveguide-integrated nonvolatile graphene *p*–*i*–*n* homojunction photodetector with other state-of-the art graphene photodetectors.

**Supplementary Table 1** Comparison of our work with the reported graphene photodetectors.

| **Structure** | **Mechanism** | **Responsivity** | ***V*_GS_ (V)**  ***V*_DS_ (V)** | **Response speed** |
| --- | --- | --- | --- | --- |
| Silicon microring integrated graphene^S3^ | PTE | 90 V/W | -0.5,-2.1  0 | 12 GHz |
| Graphene/WS_2_/WSe_2_^S4^  (vertical illumination) | Photogating | 1.7×10^7^ mA/W | /, 3 | 3-4 μs |
| Au@GQD  (vertical illumination)^S5^ | Photogating | >10^3^ A/W | /, 1 | 65 ms (rise time)  53 ms (fall time) |
| AuNRs/MoS_2_/graphene  (vertical illumination)^S6^ | Photogating | 19.49 A/W | 0, 1 | 20.5 ms (rise time)  29.5 ms (fall time) |
| Waveguide-integrated graphene  This work | PTE | 193 mA/W | 0, 0 | >17 GHz |

We compared the performance of our waveguide-integrated nonvolatile graphene *p*–*i*–*n* homojunction photodetector with previously reported graphene photodetectors, as shown in Supplementary Table 1. All of these photodetectors were proposed to provide graphene-based photodetectors. The comparisons in the characteristics of these photodetectors are described as follows: For device configuration, the P(VDF-TrFE) ferroelectric layer in our proposed waveguide-integrated nonvolatile graphene homojunction provides a nonvolatile ferroelectric polarization field, allowing the device to operate without bias or gate voltage. This P(VDF-TrFE) solution-processed method for device fabrication offers greater advantages for large-scale integration compared to other graphene photodetectors, further enhancing its practical applicability^S7,S8^. Although the responsivity of our PTE-based graphene device with zero bias and zero gate voltage is lower than that of those graphene photodetectors utilizing the photogating effect with bias and gate voltage, it consumes zero electrical energy due to zero bias and zero gate voltage and offers a significantly higher response speed. For the response speed of >17 GHz obtained in our waveguide-integrated nonvolatile graphene *p*–*i*–*n* homojunction photodetector, it is superior with other state-of-the-art graphene photodetectors. For the photoresponse spectral range, the zero bandgap of graphene allows our device to operate across the whole telecommunication band, which performs same as other graphene photodetectors.


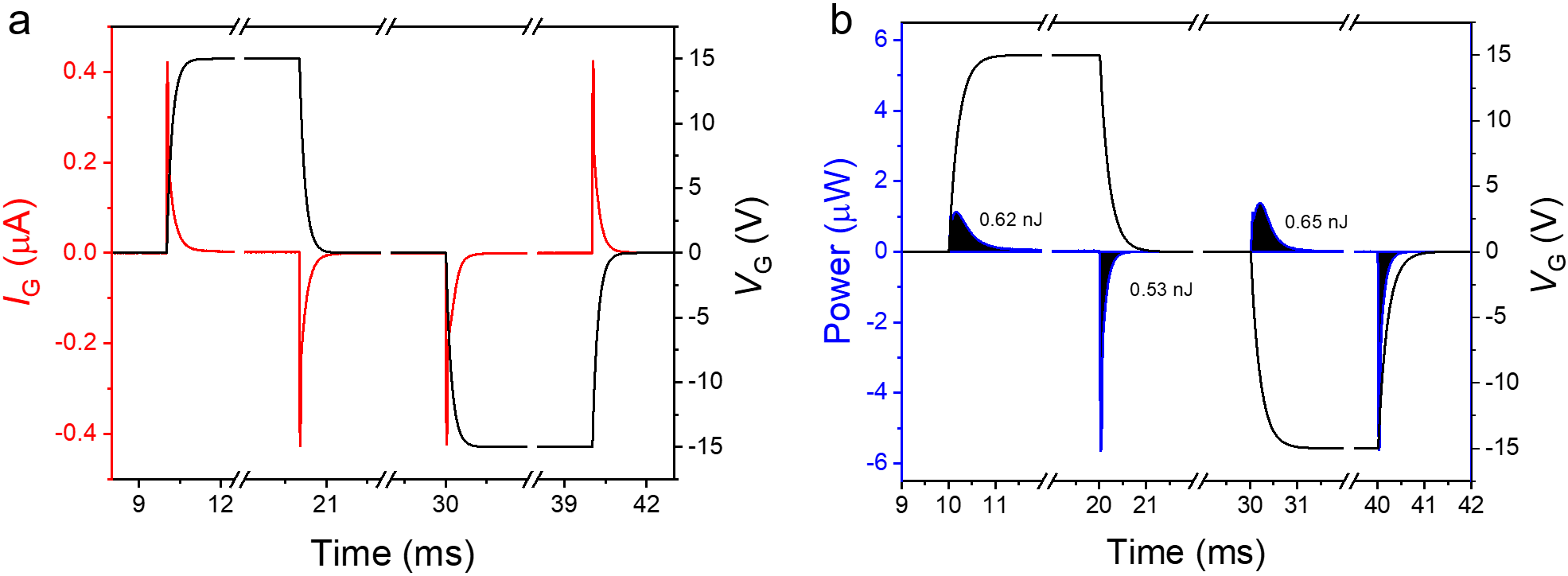


**Figure S14** **a** Measured gate current (switching transient current response) during gate pulse voltage operation. **b** Power consumption for the gate pulse voltage operation calculated from the gate current.

Our photodetector consumes zero electrical energy after programming due to the non-volatile ferroelectric ability and self-powered photogeneration process. Electrical energy consumption for the device only occurs during the application of a gate pulse voltage. To calculate the power consumption during gate application, we record the pulse (switching transient current response) current response *I*_G_ by applying a gate pulse voltage in the Au/P(VDF-TrFE)/Au capacitor, as shown in Fig. S14a. The electrical energy consumption can be calculated by the term of $\int_{\text{t1}}^{\text{t2}} \text{V}_{\text{G}}\text{I}_{\text{G}}\text{dt}$. Together with the area of the tested capacitor, we obtain an electrical energy consumption per unit area of the ferroelectric capacitor as 0.25 pJ μm^-2^. For our device, with an area of 485 μm², the total electrical energy consumption is calculated as 121 pJ, as indicated in Fig. S14b. As a result, the initial programming of the *p*–*i*–*n* junction require an electrical energy consumption of 121 pJ.

The following table provides a comparison of the waveguide-integrated nonvolatile graphene *p*–*i*–*n* homojunction photodetector with the other waveguide-integrated photodetectors across several key performance metrics.

**Supplementary Table 2** Comparison of our work with the reported waveguide-integrated photodetectors.

| **Material** | **Device structure** | **Mechanism** | **Responsivity** | ***V*_GS_ (V)**  ***V*_DS_ (V)** | **Response speed** |
| --- | --- | --- | --- | --- | --- |
| BP^S9^ | Gate tuned MSM | PV | 135 mA/W | -0.4 , -8 | 3 GHz |
| MoS_2_^S10^ | Gate tuned MSM | PG | >10^3^ A/W | 0, 1 | 13s, 11s |
| MoTe_2_^S11^ | Split gate *p*-*n* junction | PV | 4.8 mA/W | 0, 15, -15 | 200 MHz |
| Ge^S12^ | Dopped *p*-*i*-*n* junction | PV | 0.89 A/W | -2, / | 31 GHz |
| InGaAs/InAlGaAs^S13^ | Dopped *p*-*i*-*n* junction | PV | 0.78 A/W | -3, / | 28 GHz |
| Graphene-This work | Split gate *p*-*i*-*n* junction with ferroelectric layer | PTE | ~193 mA/W | 0, 0, 0 | >17 GHz |

We compared the performance of our waveguide-integrated nonvolatile graphene *p*–*i*–*n* homojunction photodetector with the other waveguide-integrated photodetectors, as shown in Supplementary Table 2. These photodetectors include waveguide-integrated BP, MoS_2_, MoTe_2_, Ge, and III-V-based photodetectors. Key characteristics of these photodetectors are summarized as follows:

For device configuration, the P(VDF-TrFE) ferroelectric layer in our proposed waveguide-integrated nonvolatile graphene homojunction provides a stable, nonvolatile ferroelectric polarization field, allowing it to operate without continuous bias or gate voltage. This feature simplifies circuit design and reduces power consumption, in contrast to other waveguide-integrated photodetectors that require a continuous voltage to maintain performance. For the response speed of >17 GHz obtained in our waveguide-integrated nonvolatile graphene *p*–*i*–*n* homojunction photodetector, it is superior to other waveguide integrated TMDCs photodetectors, due to the high carrier mobility of graphene. It is on par with the waveguide-integrated Ge, III-V compound photodetectors^S12,13^. For the responsivity of 193 mA W^-1^ obtained in our on-chip nonvolatile graphene *p*–*i*–*n* homojunction photodetector, it is on par with those of waveguide integrated TMDCs photodetectors. Although waveguide-integrated MoS_2_ photodetector has a high responsivity of 1000 A W^-1^ based on the photo-gating effect^S11^, the response time is as low as 13 s, which limits the practical applications potential. In addition, the device fabrication process of our waveguide-integrated nonvolatile graphene *p*–*i*–*n* homojunction photodetector is much simpler than waveguide integrated Ge and III-V that need complicated ion doping engineering^S12,13^.


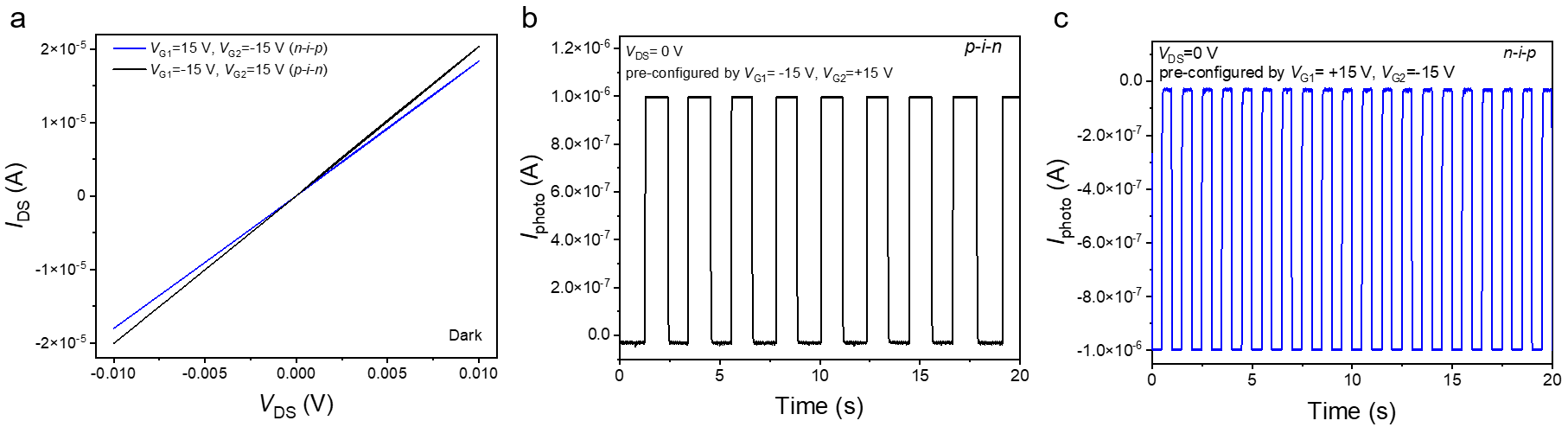


**Figure S15** **a** Output curves of the device after programming the pulse gate with *V*_G1_= −15 V, *V*_G2_= +15 V (*p*–*i*–*n*) and *V*_G1_= +15 V, *V*_G2_= −15 V (*n*–*i*–*p*). **b** *I*-*t* curve under the illumination of 1620 nm at *V*_DS_= 0 V by pre-configured pulse gate *V*_G1_= −15 V, *V*_G2_= +15 V. **c** *I*-*t* curve under the illumination of 1620 nm at *V*_DS_= 0 V by pre-configured pulse gate *V*_G1_= +15 V, *V*_G2_= −15 V.

**References**

S1. Wu, J., Xu, H. & Zhang, J. Raman spectroscopy of graphene. *Acta Chimica Sinica* **72**, 301–318 (2014).

S2. Li, C. *et al.* Waveguide-integrated MoTe_2_ p–i–n homojunction photodetector. *ACS Nano* **16**, 20946–20955 (2022).

S3. Schuler, S. *et al.* High-responsivity graphene photodetectors integrated on silicon microring resonators. *Nature Communications* **12**, 3733 (2021).

S4. Tsai, M. Y. *et al.* Ultrafast and broad-band graphene heterojunction photodetectors with high gain. *ACS Nano* **17**, 25037–25044 (2023).

S5. Thakur, M. K. *et al.* Microplasma-enabled graphene quantum dot-wrapped gold nanoparticles with synergistic enhancement for broad band photodetection. *ACS Applied Materials & Interfaces* **12**, 28550–28560 (2020).

S6. Tomar, D. S., Ghosh, S., Jhan, L. C. & Chattopadhyay, S. Gold Nanorod-Activated Graphene/MoS_2_ Nanosheet-based photodetectors for bidirectional photoconductance. *ACS Applied Nano Materials* **6**, 1783–1795 (2023).

S7. Stadlober, B., Zirkl, M. & Irimia-Vladu, M. Route towards sustainable smart sensors: Ferroelectric polyvinylidene fluoride-based materials and their integration in flexible electronics. *Chemical Society Reviews* **48**, 1787–1825 (2019).

S8. Qian, X., Chen, X., Zhu, L. & Zhang, Q. M. Fluoropolymer ferroelectrics: Multifunctional platform for polar-structured energy conversion. *Science* **380**, eadg0902 (2023).

S9. Youngblood, N., Chen, C., Koester, S. J. & Li, M. Waveguide-integrated black phosphorus photodetector with high responsivity and low dark current. *Nature Photonics* **9**, 247–252 (2015).

S10. Gonzalez Marin, J. F., Unuchek, D., Watanabe, K., Taniguchi, T. & Kis, A. MoS_2_ photodetectors integrated with photonic circuits. *npj 2D Materials and Applications* **3**, 14 (2019).

S11. Bie, Y.-Q. *et al.* A MoTe_2_-based light-emitting diode and photodetector for silicon photonic integrated circuits. *Nature Nanotechnology* **12**, 1124–1129 (2017).

S12. Yin, T. *et al.* 31 GHz Ge n-i-p waveguide photodetectors on silicon-on-insulator substrate. *Optics Express* **15**, 13965–13971 (2007).

S13. Barbara, S. & Korea, S. 40 Gbit/s waveguide photodiode using III – V on silicon heteroepitaxy. *Optics Letters* **45**, 2954–2956 (2020).
